# *In Vitro* Continuous Fermentation Model (PolyFermS) of the Swine Proximal Colon for Simultaneous Testing on the Same Gut Microbiota

**DOI:** 10.1371/journal.pone.0094123

**Published:** 2014-04-07

**Authors:** Sabine A. Tanner, Annina Zihler Berner, Eugenia Rigozzi, Franck Grattepanche, Christophe Chassard, Christophe Lacroix

**Affiliations:** Laboratory of Food Biotechnology, Institute of Food, Nutrition and Health, ETH Zurich, Zurich, Switzerland; Charité, Campus Benjamin Franklin, Germany

## Abstract

*In vitro* gut modeling provides a useful platform for a fast and reproducible assessment of treatment-related changes. Currently, pig intestinal fermentation models are mainly batch models with important inherent limitations. In this study we developed a novel *in vitro* continuous fermentation model, mimicking the porcine proximal colon, which we validated during 54 days of fermentation. This model, based on our recent PolyFermS design, allows comparing different treatment effects on the same microbiota. It is composed of a first-stage inoculum reactor seeded with immobilized fecal swine microbiota and used to constantly inoculate (10% v/v) five second-stage reactors, with all reactors fed with fresh nutritive chyme medium and set to mimic the swine proximal colon. Reactor effluents were analyzed for metabolite concentrations and bacterial composition by HPLC and quantitative PCR, and microbial diversity was assessed by 454 pyrosequencing. The novel PolyFermS featured stable microbial composition, diversity and metabolite production, consistent with bacterial activity reported for swine proximal colon *in vivo.* The constant inoculation provided by the inoculum reactor generated reproducible microbial ecosystems in all second-stage reactors, allowing the simultaneous investigation and direct comparison of different treatments on the same porcine gut microbiota. Our data demonstrate the unique features of this novel PolyFermS design for the swine proximal colon. The model provides a tool for efficient, reproducible and cost-effective screening of environmental factors, such as dietary additives, on pig colonic fermentation.

## Introduction

The pig gut microbiome is a complex ecosystem, dominated by members of the phyla Firmicutes and Bacteroidetes [1]. The vast quantity and diversity of the gut bacterial community provides the host with a large set of metabolic functions and is considered to play a key role in host health and disease [2]. Diet is a principal factor shaping the gut bacterial composition and functionality [3], thereby also impacting overall animal health. This in turn largely determines productivity and efficiency of swine livestock production. Given the complexity of this interplay, it is of paramount importance to precisely evaluate the effects of specific feed ingredients or additives on composition and functionality of the gut microbiome and further elucidate the role and function of the gut bacterial ecosystem in animal health to improve productivity and efficiency of swine livestock production.

It is obvious that animal testing is one of the most prominent strategies to predict effectiveness and impact of dietary additives on the gut microbiota, but ethical concerns and costs can restrict these applications [2]. Intestinal *in vitro* models are able to partly evade these restrictions by enabling reproducible experimentation under standardized conditions, and more importantly, giving the yet host-uncoupled opportunity to investigate the complexity of gut microbiomes and the functional relatedness of specific bacterial species [4].

To date, most porcine *in vitro* models are using simple batch cultures with the aim to examine the fermentation capacity of intestinal ecosystems on a given substrate, using the cumulative gas production technique [5]–[8]. However, batch fermentations are limited in terms of experimental duration and the amount of substrate supply to avoid negative feedback mechanisms [9]. Batch cultures are also highly dependent on the inoculation density as it directly impacts microbial growth in these closed systems [4]. In contrast, continuous culture systems are superior in modeling the dynamic nature of the gastrointestinal tract, allowing the adaptation of various parameters, including dilution rate, retention time, pH and temperature, to meet and maintain optimal growth conditions [9]. Substrate replenishment and toxic waste removal, further, is continuous and facilitates studies on the modulation of microbial composition and activity [4]. So far, only few porcine semi-continuous or continuous intestinal fermentation models inoculated with feces or cecal content have been described, focusing on changes in gut bacterial communities tested over limited fermentation periods [10], the inhibition of *Salmonella* by medium-chain fatty acids [11] or the effect of live yeast on fermentation parameters [12]. Long-term continuous *in vitro* models of the swine gut are still lacking, likely due to the difficulty of generating highly stable free-cell suspension fermentations, inoculated with fecal extract, over a long experimental period.

To overcome possible drawbacks associated with free-cell suspension cultures, such as limited stability and washout of less dominant or slow growing bacterial species, fecal microbiota was immobilized in polysaccharide gel beads to set up stable continuous intestinal fermentation models [13], [14]. The major benefits shown for immobilized fecal microbiota models include high cell density, maintenance of bacterial diversity and high stability over extended fermentation periods, tested for up to 71 days [4], [15]. Besides stability requirements, reproducibility and parallel testing of treatments with the same gut microbiota are of major importance for gut *in vitro* research, but difficult to apply with classical continuous models inoculated with fecal microbiota. We have recently set up and validated a novel PolyFermS model of the child proximal colon in a two-stage system for parallel testing of treatments on the same microbiota [16]. In this model, a first-stage reactor containing immobilized fecal microbiota and operated with conditions mimicking the first section of the proximal colon was used to constantly inoculate up to four second-stage reactors, operated with conditions mimicking the remaining proximal colon. Our data demonstrated that this PolyFermS model produced reproducible and stable intestinal microbiota over a 38 days test period [16]. The microbial diversity of reactor effluents tested with the HITChip phylogenetic array was comparable to the feces of the healthy donor, whereas a high response to pH was demonstrated. However, this study used a two-stage model for upper and lower sections of the proximal colon, shown to be highly dependent on pH in the first reactor, which had to be arbitrary set at 5.5 to reach a metabolic balance for the child microbiota.

In the present study we aimed to enhance the original design of the PolyFermS model validated with child microbiota [16], and to adapt to a new model of the swine proximal colon. The novel PolyFermS model consisted of two stages; a first stage for the inoculum reactor (IR) seeded with immobilized swine fecal microbiota and used to inoculate at 10% (v/v) five parallel second-stage reactors, which were also fed with 90% fresh chyme medium. Each reactor was operated under identical conditions, selected to mimic swine proximal colon, and allowing the parallel testing of multiple treatments on the same gut microbiota. The stability of the complex intestinal microbiota in the multiple reactors was monitored over 54 days of fermentation by analyzing its composition (qPCR and 454 pyrosequencing) and metabolic activity (HPLC).

## Material and Methods

### Ethical statement

No specific permits were obtained for the collection of the fecal sample. The animal was not harmed during fecal sample collection and oral consent for sample collection was obtained from the owner of the farm.

### Feces collection and immobilization

Feces from a healthy 5 month old sow (80 kg), raised under farming conditions and not subjected to any antibiotic treatment for the last 3 months, were collected in a sterile 50 mL Falcon tube. Anaerobiosis was maintained using anaerobic gas pack systems (Oxoid AnaeroGen TM, Oxoid AG, Basel Switzerland) during transport to the laboratory and until immobilization was performed. The entire immobilization procedure was carried out under anaerobic conditions (anaerobic chamber; Coy Laboratories, Ann Arbor, MI, USA). Briefly, a 20% (w/v) suspension of feces in pre-reduced peptone water (0.1%, pH 7) was prepared, homogenized and further immobilized in a polymer solution consisting of gellan gum (2.5%, w/v), xanthan (0.25%, w/v) and sodium citrate (0.2%, w/v) for production of 1–2 mm diameter gel beads using a two phase dispersion process as described previously [13], [16].

### Nutritive medium

The nutritive medium described by Macfarlane *et al.* [17] was modified for its carbohydrate and protein concentration to more closely mimic the ileal chyme of a swine (Table S1), using a similar approach as described previously [15]. For calculation of ingredient concentrations, a standard cornstarch based diet with corn (641 g kg^−1^) as main carbohydrate and soybean meal (331 g kg^−1^) as main N-source was used [18]. Digestibility indices of 97% for cornstarch [19] and 82.5% for soybean meal [20] were applied while considering a 2 kg/day feed intake per pig, resulting in a cornstarch:N-compound (soybean meal) ratio of 25:75. The amount of cornstarch and soy peptone supplied daily to the model was calculated by applying a scale factor of 0.09 to account for the actual volume of the proximal colon *in vivo* (approx. 2.9 L [21] compared to the proximal reactor volume of the model (260 mL). The final concentration of the two compounds in the chyme medium was estimated for a mean retention time of 9 hours in the reactors, giving a daily chyme medium supply per reactor of 693 mL. To avoid excess of nitrogen compounds, the soy peptone concentration was fixed to 13 g L^−1^ resulting in a carbohydrate (cornstarch) concentration of 4.3 g L^−1^. Yeast extract and mucin, mimicking the contribution of endogenous secretion [13], and the non-starch polysaccharides (pectin, xylan, arabinogalactan and guar gum) were excluded from this calculation.

A volume of 0.5 mL L^−1^ of a filter-sterilized (Minisart pore size 0.2 μm, Sartorius, VWR International AG, Dietikon, Switzerland) vitamin solution described by Michel *et al.* [22] was added to the sterilized medium (20 min, 120°C). All components of the nutritive medium were purchased from Sigma-Aldrich Chemie (Buchs, Switzerland), except for soy peptone (Labo-Life Sàrl, Pully, Switzerland), yeast extract (I2CNS GmbH, Urdorf, Switzerland) and KH_2_PO_4_ (VWR International AG).

### Experimental set-up of the PolyFermS model

**Figure 1:**
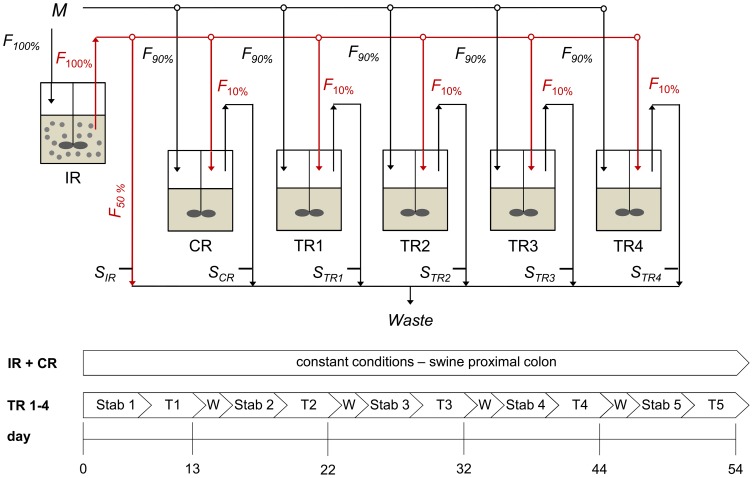
Experimental reactor set-up and time schedule of the swine PolyFermS model. IR: inoculum reactor, containing immobilized swine feces (30% v/v); CR: control reactor; TR1-TR4: test reactors 1-4; *M*: fresh nutritive medium supply; *S*: effluent sampling; *F*: flow rate; Stab: stabilization period; T: treatment period; W: wash period

The continuous fermentation was carried out for 54 days using a two-stage design with a total of six reactors (Sixfors, Ismatec, Glattbrugg, Switzerland) (Figure 1). Each reactor was aimed to simulate conditions of the swine proximal colon fermentation.

The inoculum reactor (234 mL) was seeded with 30% (v/v) swine fecal beads and connected via a peristaltic pump (Masterflex L/S, Fisher Scientific SA, Wohlen, Switzerland) to one control reactor (CR) and four test reactors (TR1-4) operated in parallel. IR was supplied with 100% fresh nutritive medium (26 mL h^−1^) whereas the second-stage reactors CR and TR1-4 were continuously supplied with 90% (26 mL h^−1^) fresh nutritive medium and 10% (2.9 mL h^−1^) effluent from IR for continuous inoculation. The remaining effluent (50%) from IR was discarded. The inoculation rate of CR and TR1-4 was accurately controlled using an in-house designed distributor device equipped with valves regulated chronometrically.

### Fermentation procedure

The PolyFermS model was run under conditions of the swine proximal colon with a controlled constant pH of 6.0 through addition of 2.5 M NaOH. The mean retention time was fixed at 9 hours, while temperature was maintained at 38°C (being in the middle of the range previously applied in porcine *in vitro* models) [7], [10], [11], [23]–[25]. Anaerobic conditions were ensured by constantly flushing the headspace of the reactors with CO_2_ and constant stirring was performed at 120 rpm. During the first 72 hours, IR was operated in batch mode to colonize the fecal beads and the nutritive medium was replaced by fresh medium every 12 hours. After colonization, continuous operation (26 mL h^−1^) in IR was started, followed by a stabilization period of 5 days before connection to CR and TR1-4. The entire two-stage system was then stabilized for another 5 days.

The 54 days continuous fermentation was split into stabilization, treatment and washing periods (Figure 1). IR and CR were operated with constant conditions to assess the temporal stability of the system and were not subjected to any manipulation during the entire fermentation. In addition, CR served as a control reactor for TR1-4 that were subjected to different parallel treatment periods (data not shown).

Between two treatment periods, TR1-4 were subjected to a washing procedure with 10% chlorine to kill microbes and remove any historical effect of the previous periods. Briefly, TR1-4 were disconnected from IR, the entire medium was removed and reactors were filled with 10% freshly prepared chlorine solution. After stirring for one hour, the reactors were rinsed twice by adding sterile bidistilled water and stirring for another hour. After complete removal of water and chlorine residues, reactors were filled with sterile fresh nutritive medium and reconnected to IR. Thereafter, the system was allowed to stabilize for 3 days until reaching steady state before starting the next treatment period.

Effluent samples of all reactors were collected daily. HPLC samples were processed immediately whereas samples for DNA extraction were stored at −80°C.

### HPLC analysis for metabolite determination

Short-chain fatty acids (SCFA; acetate, propionate, butyrate, valerate, formate, *iso-*butyrate and *iso*-valerate) as well as lactate concentrations in fermentation effluent samples from all reactors were determined by HPLC analysis (Thermo Fisher Scientific Inc. Accela, Wohlen, Switzerland). Briefly, effluent samples were centrifuged (14 000 g) for 10 min at 4°C. The pellet was used for DNA extraction while the supernatant was diluted 1∶10 with ultrapure water and filtered directly into vials through a 4 mm HPLC filter with a 0.45 μm nylon membrane (Infochroma AG, Zug, Switzerland). The analysis was run at a flow rate of 0.4 mL min^−1^ using an Aminex HPX-87H column (Bio-Rad Laboratories AG, Reinach, Switzerland) and 10 mM H_2_SO_4_ as eluent. Mean metabolite concentrations were calculated from duplicate analyses and expressed in mM.

### DNA extraction and qPCR analyses

Genomic DNA was extracted from effluent samples using the FastDNA SPIN Kit for soil (MP Biomedicals, Illkirch, France). DNA extracts were subjected to quantitative real-time PCR (qPCR) for enumeration of specific bacterial target groups comprising total bacteria, *Bifidobacterium* spp., *Bacteroides*-*Prevotella* group, *Enterobacteriaceae*, *Lactobacillus/Pediococcus/Leuconostoc* spp. and *Clostridium* Cluster IV (Table 1). In addition, the *Succinivibrio dextrinosolvens* group was quantified to verify results obtained by 454 pyrosequencing. Standard curves for each target group were prepared as described previously [26]. All assays, were performed using the 2 x SYBR Green PCR Master Mix (Applied Biosystems, Zug, Switzerland) in a 25 μl volume and an ABI PRISM 7500-PCR sequence detection system (Applied Biosystems).

**Table 1:** Primers for detection of specific bacterial groups by qPCR.

Target	Primer	Sequence 5′-3′	Reference
Total 16S rRNA genes	Eub338F	ACT CCT ACG GGA GGC AGC AG	[48]
	Eub518R	ATT ACC GCG GCT GCT GG	
*Bacteroides*-*Prevotella* group	Bac303F	GAA GGT CCC CCA CAT TG	[49]
	Bfr-Fmrev	CGC KAC TTG GCT GGT TCA G	
*Lactobacillus*/*Pediococcus*/*Leuconostoc* spp.	F_Lacto 05	AGC AGT AGG GAA TCT TCC A	[50]
	R_Lacto 04	CGC CAC TGG TGT TCY TCC ATA TA	
*Enterobacteriaceae*	Eco1457F	CAT TGA CGT TAC CCG CAG AAG AAG C	[51]
	Eco1652R	CTC TAC GAG ACT CAA GCT TGC	
*Bifidobacterium* spp.	xfp_fw	ATC TTC GGA CCB GAY GAG AC	[52]
	xfp_rv	CGA TVA CGT GVA CGA AGG AC	
*Clostridium* Cluster IV	Clep866mF	TTA ACA CAA TAA GTW ATC CAC CTG G	[49]
	Clep1240mR	ACC TTC CTC CGT TTT GTC AAC	
*S. dextrinosolvens* group	SucDex1F	CGTCAGCTCGTGTCGTGAGA	[53]
	SucDex1R	CCCGCTGGCAACAAAGG	

### 454 pyrosequencing

Selected samples were analyzed by 454 pyrosequencing for their microbial 16S rRNA based composition profile. IR and CR (days 19/20, 30/31, 42/43 and 52/53) samples were selected to assess bacterial diversity and temporal stability of the model as well as to compare the microbial composition to the fecal inoculum. To demonstrate the re-establishment of the microbiota after washing, day 25 (last day of 3^rd^ stabilization period) was chosen as a representative day for all reactors. For IR and CR stability samples, effluent from 2 consecutive days were pooled at a ratio of 1∶1. DNA was extracted with the FastDNA Spin Kit for soil (MP Biomedicals) and sent to DNAVision SA (Charleroi, Belgium) for 454 pyrosequencing analysis and subsequent taxonomic assignment of 16S rRNA gene reads. 454 pyrosequencing was performed using a 454 Life Science system combined with Titanium Chemistry (Roche) as described previously [27]. The complete 454 pyrosequencing dataset has been deposited to the National Center for Biotechnology Information (NCBI) Sequence Read Archive (SRA) under accession number SRP034540.

### Statistical analysis

All statistical analyses were performed using PASW Statistics for Windows version 18.0 (SPSS Inc., Chicago). qPCR data were log10-transformed and expressed as means ± SD of the last three days of each stabilization period. To assess reproducibility of the microbial composition in CR and TR1-4 prior to a treatment period qPCR data were subjected to the non-parametric Mann-Whitney U test with exact significance and a p-value <0.05 was considered significant. The same test was used to verify results obtained from 454 pyrosequencing for *Succinivibrionaceae*. Comparisons were made between qPCR data from before (d11-37) and after (d38-54) appearance in CR. To assess temporal metabolite stability in IR and CR, linear regression of total SCFA, acetate, butyrate, propionate, *iso-*valerate, valerate and *iso-*butyrate concentrations versus time were calculated over the time period d11-54 and difference from 0 of slope coefficients was tested using the t-test (*P<0.05*).

## Results

### Microbial activity by HPLC

To assess the microbial activity and temporal stability of the novel PolyFermS model for the pig proximal colon during 54 days continuous fermentation, daily effluents for each reactor were analyzed using HPLC. IR and CR were operated with constant conditions and not subjected to any treatment or washing period during the entire fermentation. Therefore, the microbial activity in these two reactors was used to assess the metabolic stability of the two-stage model.

**Figure 2:**
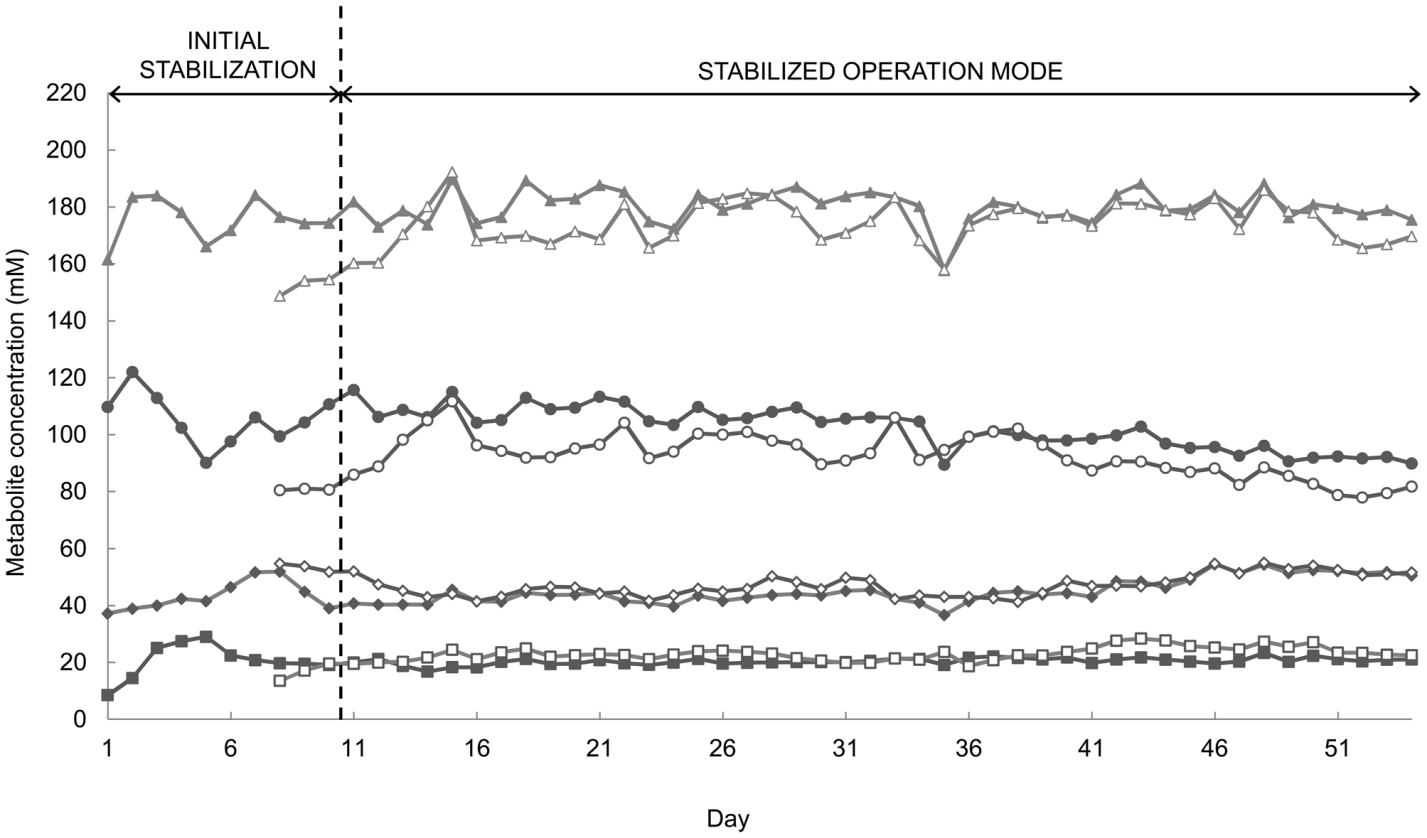
Daily main SCFA concentrations in fermentation effluents of IR and CR measured by HPLC. Initial stabilization: stabilization period in continuous mode to reach pseudo steady-state; closed symbol: IR; open symbol: CR; (▴, Δ) total SCFA, (•, ○) acetate, (♦, ◊) propionate, and (▪, □) butyrate.

After an initial stabilization period of 10 days for reaching pseudo-steady state conditions, high and stable metabolic activity was measured in IR and CR throughout the fermentation (Figure 2). In IR the mean total SCFA concentration (d11-54) was 180.1±5.8 mM with mean values for acetate, propionate and butyrate of 102.3±7.3 mM, 45.1±4.4 mM and 20.4±1.2 mM, respectively. *Iso-*valerate was produced at 5.9±0.5 mM. In contrast, valerate and *iso-*butyrate were not detected until day 13 but were measured at concentrations of 4.5±1.3 mM and 1.9±0.6 mM over d13-54, respectively. Lactate and formate, were not detected throughout the fermentation. For CR similar mean total SCFA (174.4±7.6 mM) as well as main SCFA acetate (92.9±7.6 mM), propionate (47.1±3.8mM) and butyrate (23.1±2.4 mM) concentrations were detected. Detection of minor metabolites, *iso-*valerate, valerate and *iso*-butyrate, was delayed for 9, 14 and 13 days in CR, respectively, resulting in mean values of 5.7±1.2 mM (d11-54), 4.2±1.6 mM (d14-54) and 1.8±0.9 mM (d13-54).

To test the effect of culture time on metabolite concentrations, linear regressions of daily concentration data (d11-54) versus time were calculated. Highly significant time effects (*P<*0.001) were determined for most measured metabolites in IR and CR, except for the total SCFA concentration (Table 2). Acetate concentrations in IR and CR (*P*<0.001) and butyrate in CR (*P*<0.05) decreased significantly over time whereas propionate, butyrate in IR, *iso*-valerate, valerate and *iso*-butyrate showed significant (*P*<0.001) concentration increases. Corresponding slope coefficients remained small (-0.493 mM/day – 0.265 mM/day) indicating a moderate time effect on tested metabolites.

**Table 2:** Effect of culture time on metabolite concentrations analyzed by linear regression analysis.

	Inoculum reactor (IR)			Control reactor (CR)		
	B^a^	SE B	R^2^	B^a^	SE B	R^2^
total SCFA	−0.030	0.069	0.005	0.092	0.090	0.024
acetate	−0.493^**^	0.043	0.760	−0.365^**^	0.072	0.376
propionate	0.265^**^	0.034	0.597	0.168^**^	0.038	0.317
butyrate	0.050^**^	0.012	0.292	−0.090^*^	0.025	0.238
*iso*-valerate	0.031^**^	0.004	0.544	0.063^**^	0.010	0.486
valerate	0.080^**^	0.009	0.637	0.093^**^	0.013	0.542
*iso*-butyrate	0.037^**^	0.005	0.593	0.047^**^	0.007	0.509

a slope coefficients significantly different from 0 are denoted by significance level:^*^ *P<0.05* ^**^ *P<0.001.*

B: unstandardized (slope) coefficient (mM/day); SE B: Standard error of B; R^2^: coefficient of determination.

### Microbial composition by qPCR

Microbial composition of daily reactor effluents was assessed by analyzing the 16S rRNA gene copy numbers of bacterial target groups using qPCR. IR and CR data were used to assess the time stability of the model.

Total 16S rRNA gene copy numbers mL^−1^ effluent were approximately 1.6 log_10_ units higher in the fecal inoculum compared to the reactor effluents from IR and CR (Table 3). The fecal inoculum was dominated by the *Bacteroides-Prevotella* group and the *Lactobacillus/Pediococcus/Leuconostoc* spp. group, followed by *Clostridium* Cluster IV, *Enterobacteriaceae* and *Bifidobacterium* spp. In reactor effluents from IR and CR, stable copy numbers of the targeted bacterial groups were recorded during continuous fermentation (d11-54) after an initial stabilization as indicated by low standard deviations of the mean. Similar to the fecal inoculum, *Bacteroides-Prevotella* group was predominant in IR and CR effluents while *Bifidobacterium* spp. was the least abundant group. *Enterobacteriaceae* were approximately 1.8 log_10_ higher in reactor effluents compared to the fecal inoculum and displayed equally high copy numbers as for the *Clostridium* Cluster IV. In contrast, the *Lactobacillus/Pediococcus/Leuconostoc* spp. population was detected at ca. 3 log_10_ lower copy numbers in reactor effluents during the stabilized period (d11-54) compared to the fecal inoculum.

**Table 3:** Mean concentration (log_10_ copy numbers mL^−1^ effluent) of specific bacterial groups measured by qPCR in the fecal inoculum and effluent samples from inoculum reactor (IR) and control reactor (CR) during the stabilized period (d11-54).

	fecal inoculum	IR	CR
total 16S rRNA gene	12.2	10.6±0.2	10.6±0.2
*Bifidobacterium* spp.	7.8	6.6±0.7	6.3±0.5
*Bacteroides-Prevotella* group	11.3	10.2±0.2	10.2±0.2
*Enterobacteriaceae*	8.1	9.9±0.2	9.8±0.2
*Lactobacillus/Pediococcus/Leuconostoc* spp.	11.2	8.3±0.3	8.0±0.3
*Clostridium* Cluster IV	10.7	9.9±0.2	9.9±0.2

Mean values ± SD for IR and CR were calculated from daily values during the experimental stabilized period corresponding to d11-54.

### Microbial diversity by 454 Pyrosequencing

Microbial diversity and composition analyses of the fecal inoculum and selected samples from IR and CR (days 19/20, 30/31, 42/43, 52/53) were performed by 454 pyrosequencing. Sequences were aligned with the RDP classifier v 2.1 using a confidence cutoff level of 80%. After quality check the number of reads per sample was decreased from 11661±2758 to 7762±4585 and mean read length per sample was 256±2 base pairs (bp).

**Figure 3:**
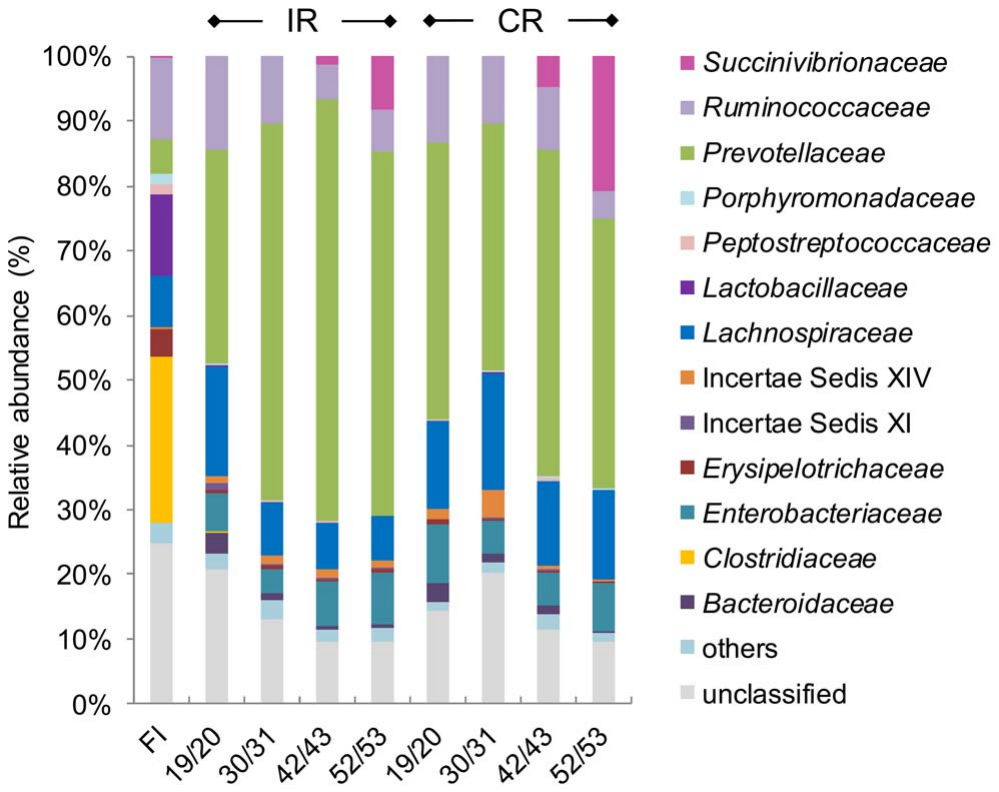
Microbial composition in the fecal inoculum (FI), IR and CR measured by 454 pyrosequencing. The relative abundance on family level is shown. Values <1% are summarized in the group "others".

Relative abundance detected by 454 pyrosequencing revealed the predominance of three major phyla in all samples tested (Figure S1A). The fecal inoculum was predominated by the phylum Firmicutes whereas the Bacteroidetes phylum was most abundant in reactor effluents, followed by the Firmicutes and Proteobacteria phyla, except for samples 19/20 in IR and 30/31 in CR where the two phyla Bacteroidetes and Firmicutes were almost equally abundant. The phylum Proteobacteria was increasing from less than 1% relative abundance in the fecal inoculum to up to 29% in sample 52/53 from CR. At family level (Figure 3), the highest abundance in IR was recorded for *Prevotellaceae* (33–66%), *Lachnospiraceae* (7–17%), *Ruminococcaceae* (5–14%) and *Enterobacteriaceae* (4–8%), with unclassified reads accounting for 10–21% of total reads. The same pattern of relative abundances on family level was observed in CR, with *Prevotellaceae* (38–51%), *Lachnospiraceae* (13–18%), *Ruminococcaceae* (5–13%) and *Enterobacteriaceae* (5–9%) being the most abundant families in all samples and unclassified bacteria accounting for 10–20% of the reads. The detected families were composed of the predominant genera *Prevotella, Escherichia*/*Shigella, Ruminococcus, Roseburia, Blautia, Bacteroides* and *Oscillibacter* (Figure S1B). Remarkably, the family *Succinivibrionaceae* increased from 0.1% on day 19/20 in IR to up to 8% at the end of the fermentation (52/53). In CR the family *Succinivibrionaceae* even accounted for as much as 21% of total reads on day 52/53. In contrast, reads assigned to the family *Ruminococcaceae* decreased in CR from 10% on day 42/43 to 4% on day 52/53. This observation was confirmed by qPCR with specific primers targeting the 16S rRNA gene of *S. dextrinosolvens.* Similarly, the 16S rRNA gene copy numbers for the *S. dextrinosolvens* group in CR significantly increased (*P*<0.001) from 7.47±0.2 log_10_ copies mL^−1^ (mean ± SD; d11-37) to 9.2±0.3 log_10_ copies mL^−1^ (mean ± SD; d38-54).

In the fecal inoculum, the family *Clostridiaceae* (26%) was most abundant and predominantly represented by the genus *Clostridium*, followed by *Ruminococcaceae* and *Lactobacillaceae* (both 13%) and *Lachnospiraceae* (8%). The unclassified bacteria accounted for 25% of total reads. *Enterobacteriaceae* were not detected in the fecal inoculum.

### Reproducibility of microbial composition and activity

**Figure 4:**
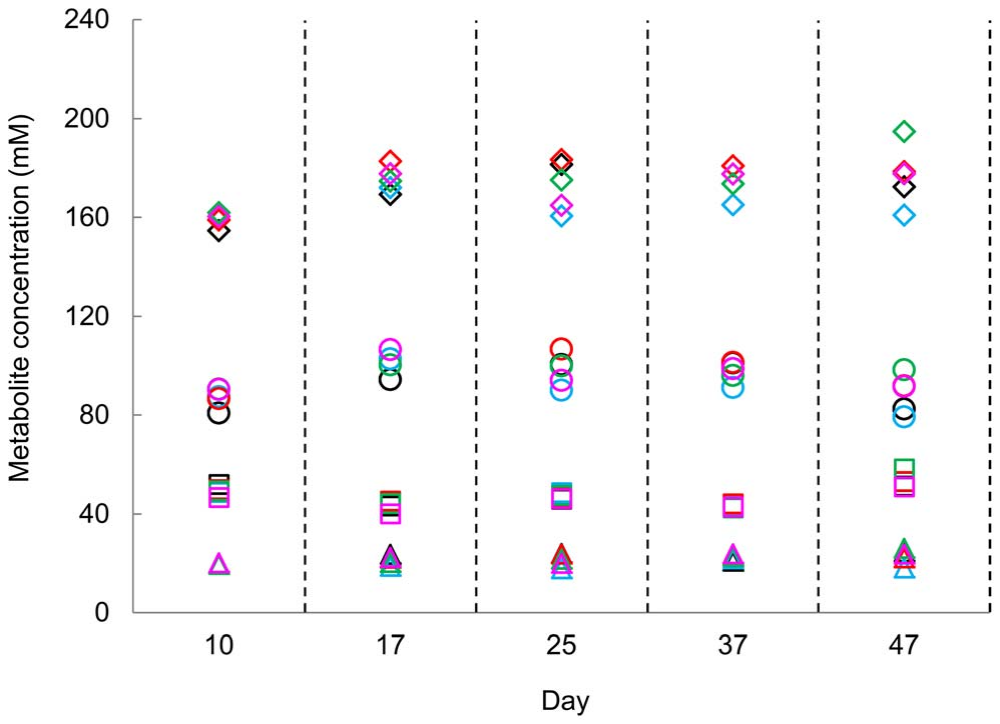
Main metabolites in CR and TR1-4 on the last day of each stabilization period. (◊) total SCFA; (○) acetate; (□) propionate; (Δ) butyrate; (black) CR; (blue) TR1; (red) TR2; (green) TR3; (pink) TR4.

The novel PolyFermS model was designed to allow reproducible testing of different treatments in parallel test reactors compared to a control reactor inoculated with the same microbiota. Consecutive multiple treatment periods could be tested by subjecting the test reactors to a washing procedure using 10% chlorine and followed by a stabilization period to regenerate comparable microbiota in CR and all test reactors (Figure 1). Therefore, to test reproducibility of control and test reactors after a washing procedure, bacterial composition (qPCR data) and activity (HPLC data) in TR1-4 after three day re-stabilization were compared with data measured in CR. No significant differences of bacterial composition of CR and TR1-4 (*P<0.*05) were detected using the Mann-Whitney U test (Table S2). Moreover the analysis of effluent samples of CR and TR1-4 by 454 pyrosequencing on day 25 (last day of 3^rd^ stabilization period) showed similar microbiota composition (Figure S2). Metabolite concentrations in TR1-4 progressively re-established at similar levels to that in CR, reaching comparable values three days after restarting the system (Figures 4 and S3). High and stable bacterial concentrations were measured already after one day re-stabilization as indicated by total 16S rRNA gene copy numbers in TR1-4 over three days stabilization periods.

## Discussion

The large field of research to evaluate the impact of feed ingredients and additives on the gastrointestinal health and development of pigs [28] emphasizes the need for potent porcine *in vitro* fermentation models to study feed-related impacts on gut microbial community and functionality in a fast and reproducible setting.

In this study we tested a novel porcine *in vitro* fermentation model for the proximal colon, advanced from the PolyFermS model presented by Zihler Berner *et al.* [16]. The porcine *in vitro* model was designed to generate self-contained parallel fermentations in each of the reactors, simulating the proximal colon, which is the primary site of fermentation in the gastrointestinal tract [29]. The first PolyFermS model validated by Zihler Berner *et al.* [16] included an inoculum reactor operated under conditions of the first section of the proximal colon which was used to continuously feed control and test reactors with fermented effluent, operated with conditions selected to mimic the distal section of the proximal colon. Therefore in this model a combination of two stages, IR and CR or TR, was used to model the proximal colon. In contrast, test reactors in the porcine PolyFermS tested in this study were operated in conditions of the proximal colon and constantly inoculated with only 10% fecal microbiota produced in IR, while 90% of the feed was fresh medium to simulate the dynamic process of chyme inflow. This design is likely more suitable for studying the fate of dietary treatments on the fermentative capacity of the gut microbial community, since the complete response of the proximal colon microbiota, constantly supplied with fresh substrates from the small intestine, is of interest. In our study, the combination of the reactor set-up and the nutritive medium, adapted to simulate swine chyme, allowed the establishment of six self-contained parallel fermentations for the porcine proximal colon with a high metabolic and compositional stability, diversity and reproducibility throughout 54 days of fermentation.

The stable metabolite concentrations obtained during the continuous fermentation, indicate balanced microbial growth and the maintenance of gut microbiota functional capacity. In addition, metabolite ratios for IR and CR (61∶27∶12 and 57∶29∶14, acetate:propionate:butyrate) after initial stabilization were very similar and also in agreement with values reported for the pig proximal colon (60∶25∶15 [30]) and for fecal SCFA ratios (63∶25∶12 [31]) *in vivo*. Small but significant time effects were recorded for all detected metabolites, except total SCFA concentrations that remained constant during the entire stabilized fermentation. The ability to detect even small time changes is directly related to the sensitivity of the analysis, permitted by continuous operation of a stabilized fermentation system and achieved with a high number of time points analyzed [32].

The slight but significant increase of propionate concentrations during continuous fermentation may directly be related to the observed increase of the family *Succinivibrionaceae,* which was already detected in the fecal inoculum but at a low relative abundance of 0.1% by 454 pyrosequencing. *Succinivibrionaceae* belong to the γ-subclass of the phylum Proteobacteria and play an important role in starch digestion in sheep and cattle [33]. While Kim *et al*. [34] assigned the genus *Succinivibrio* to the group of the less abundant genera in the swine fecal microbiome, other studies in contrast have grouped the genus as a member of the core microbiota of the porcine proximal colon [35] or porcine cecum [36]. Different carbohydrate sources can be metabolized by *Succinivibrionaceae* resulting in the main fermentation products acetate and succinate [33]. Further decarboxylation of succinate can lead to propionate [37], likely due to cross-feeding reactions in the complex intestinal environment.

Microbial composition and diversity determined in the model effluents by qPCR and 454 pyrosequencing showed no major changes in the bacterial groups between days 11 and 54 in both, IR and CR, after the initial stabilization period of 10 days. The establishment of a microbial pseudo steady-state is an important factor to gain reliable data on the modulating potential of a specific treatment in order to avoid false positive conclusions related to the microbiota adaptation to *in vitro* conditions [38]. Compared to the fecal inoculum all bacterial groups targeted by qPCR were reduced in reactor effluents from IR and CR, except for *Enterobacteriaceae* that displayed higher copy numbers. Changes in microbiota composition and diversity may reflect the transfer from *in vivo* (feces) to *in vitro* (proximal colon) conditions, the adaptation to a new environment, which depends on the conditions in the host during collection of the fecal sample, and the lack of host effects *in vitro* [16], [39]. Additionally, the shift in microbial composition may favor more robust species due to a competitive advantage in adaptation and may open niches, possibly occupied by *Enterobacteriaceae*, which can explain their increase. Using 454 pyrosequencing, *Clostridiaceae* exhibited the most remarkable decrease in relative abundance between the fecal inoculum and reactor effluents, whereas *Prevotellaceae* increased markedly in reactor effluents. The high prevalence of *Prevotellaceae* was directly linked to an increased Bacteroidetes and decreased Firmicutes ratio in reactor effluents compared to the fecal inoculum. The high occurrence of Bacteroidetes in intestinal *in vitro* models, has already been reported previously in the Twin-SHIME [39] and TNO intestinal model [40] and may be the result of the higher micromolar levels of oxygen and the less adhesive capacity of Bacteroidetes.

Studies on the swine fecal and cecal microbiome reported Firmicutes, Bacteroidetes and Proteobacteria as the most prevalent phyla in swine [1], [34], [36], [41]–[43], which is in accordance with our study. Firmicutes and Bacteroidetes represented between 80–90% of assigned reads in all effluent samples, except for CR on day 52/53, when Proteobacteria increased to 29% and Firmicutes*/*Bacteroidetes decreased to 70%, respectively. On family level, *Prevotellaceae* was the most abundant family detected in all reactor samples, which is in accordance with other studies [1], [35], [36], [41], [44] and supported by our qPCR data. On genus level the predominant genera in the porcine PolyFermS (mean relative abundance in IR and CR >1%) were *Prevotella, Escherichia/Shigella, Roseburia, Ruminococcus, Oscillibacter, Succinivibrio, Blautia* and *Bacteroides*, genera that have previously been described as members of the cecal [36], [43], [45] and fecal [1], [44] gut microbiota. We thus conclude that the microbial composition is representative for the porcine proximal colon, as demonstrated by the high accordance of bacterial genera between our *in vitro* study and previous *in vivo* studies on the cecal porcine microbiota. Furthermore, a dominant fraction of glycan degraders was identified, consisting of the predominant genera *Prevotella*, *Ruminococcus* and *Roseburia*. The high prevalence of fibrolytic bacteria and their associated carbohydrate utilization systems in swine has already been reported previously [1], [46] and is possibly related to the high amount of complex polysaccharides found in the pig diet. Starch degraders play a key role in gut microbial ecosystems as they represent the top of the trophic chain by providing simple sugars from the breakdown of complex carbohydrates, thus directly rendering them accessible to other members of the microbial community [47].

Assessing the reproducibility of *in vitro* gut fermentation models is a permanent challenge [4] and is difficult to achieve with classical models. In this study, we used a constant 10% inoculation rate from IR to the subsequent reactors, which allowed reproducing similar and parallel evolving self-contained ecosystems in IR and the second-stage reactors. Due to the consecutive treatment periods with in-between washing of the test reactors, a fast re-establishment of reproducible environment in the test reactors was required. This is of particular importance for studying and more importantly comparing treatment-related responses of the microbial community *in vitro,* which presumes comparable experimental conditions in the different reactors. While the bacterial groups in TR1-4 targeted by qPCR reached similar numbers to that of CR after only one day of re-stabilization, complete metabolic activity was recovered after a longer time of approximately three days. Such delay of functionality response has also been reported with the Twine-SHIME model [39]. The short re-stabilization period of PolyFermS compared to 5–8 days for Twin-SHIME points on the benefits of immobilized fecal microbiota to provide bacterial stability and diversity [4].

To conclude, in the present study we validated a novel PolyFermS continuous intestinal fermentation model of the swine proximal colon, inoculated with immobilized fecal microbiota. This model operated with a nutritive medium designed to mimic pig chyme allowed to stably reproduce the microbiota and metabolic activity of swine proximal colon for at least 54 days. The particular model set-up allows comparing different treatments and a control, run with the same inoculated microbiota, simultaneously. Furthermore, our data demonstrate a considerable interplay between functionality and taxonomic composition and highlight the stringent potential of the model for compositional as well as functionality related studies. This *in vitro* gut model can further be expanded to simulate multiple stages of the large intestine (proximal, transverse, distal) and a number of consecutive treatment periods. It should be particularly suitable to accurately investigate the effects of dietary factors such as pro-and prebiotics, as well as environmental parameters or drugs on the porcine gut microbiota in highly controlled settings.

## Supporting Information

Figure S1 **Microbial composition in the fecal inoculum (FI), IR and CR measured by 454 pyrosequencing on (A) phylum level and (B) genus level.** Values <1% are summarized in the group “others”.
(TIF)

Figure S2 **Microbial composition on family level in CR and TR1-4 on day 25 (last day of 3^rd^ stabilization period) measured by 454 pyrosequencing.** Values <1% are summarized in the group “others”.
(TIF)

Figure S3 **Mean main metabolite concentrations and total 16S rRNA gene copy numbers from TR1-4 during the last three days of each stabilization period.** Data are depicted as mean values ± SD from TR1-4. (♦) total 16S rRNA gene copies mL^−1^ effluent; (○) acetate; (□) propionate; (Δ) butyrate.
(TIF)

Table S1 **Composition of the nutritive medium simulating the swine ileal chyme.**
(PDF)

Table S2 **16S rRNA gene copy numbers (log_10_ mL^−1^ of effluent) of specific bacterial groups measured by qPCR in effluent samples from CR and TR1-4.**
(PDF)
