# Supplementary figures and images for: In Vitro Continuous Fermentation Model (PolyFermS) of the Swine Proximal Colon for Simultaneous Testing on the Same Gut Microbiota
Source: PLoS One. 2014 Apr 7;9(4):e94123. doi: 10.1371/journal.pone.0094123 (PMC3978012; doi:10.1371/journal.pone.0094123)

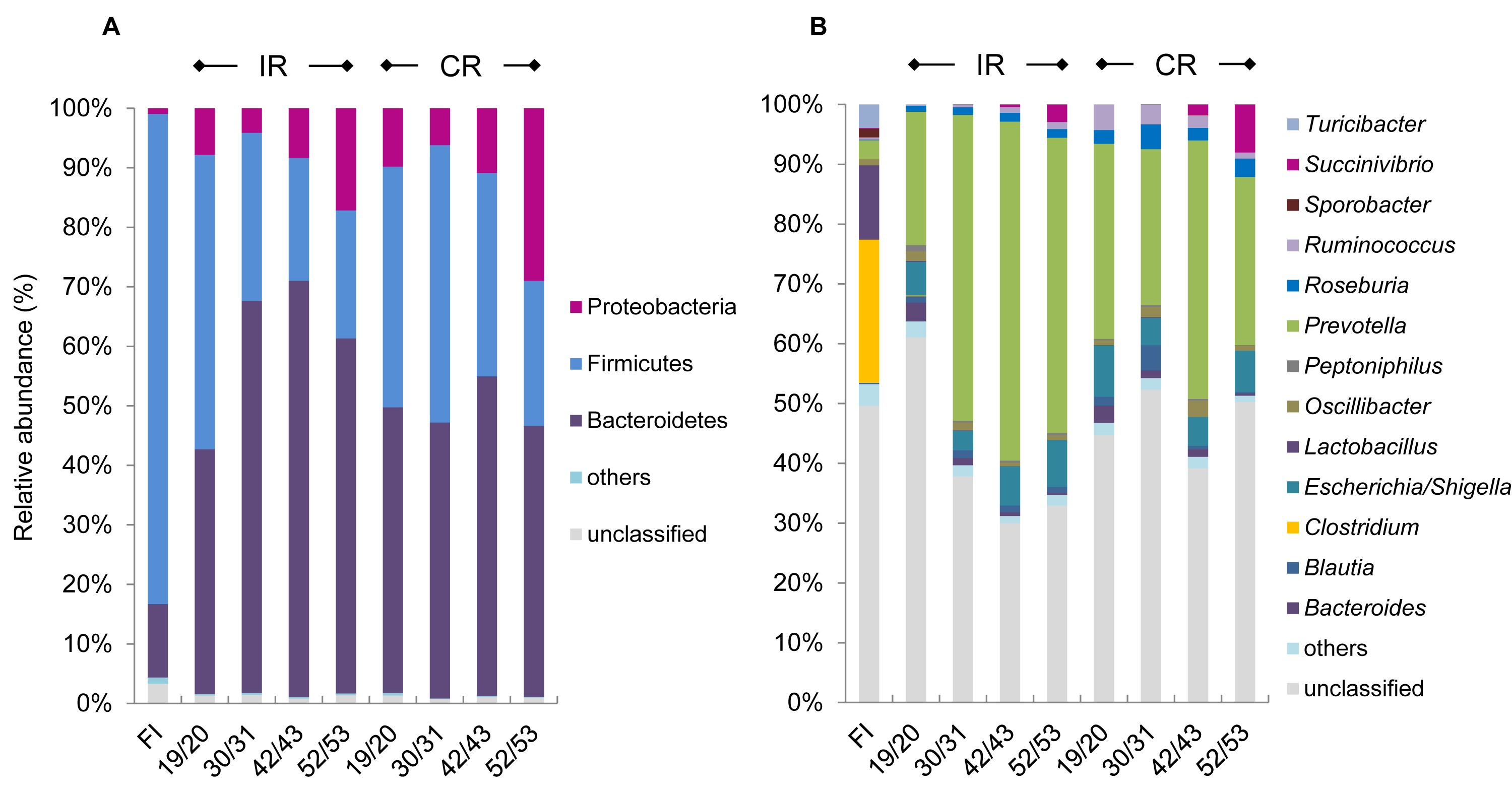

Supplement: Figure S1 — Microbial composition in the fecal inoculum (FI), IR and CR measured by 454 pyrosequencing on (A) phylum level and (B) genus level. Values <1% are summarized in the group “others”. (TIF) [file pone.0094123.s001.tif]

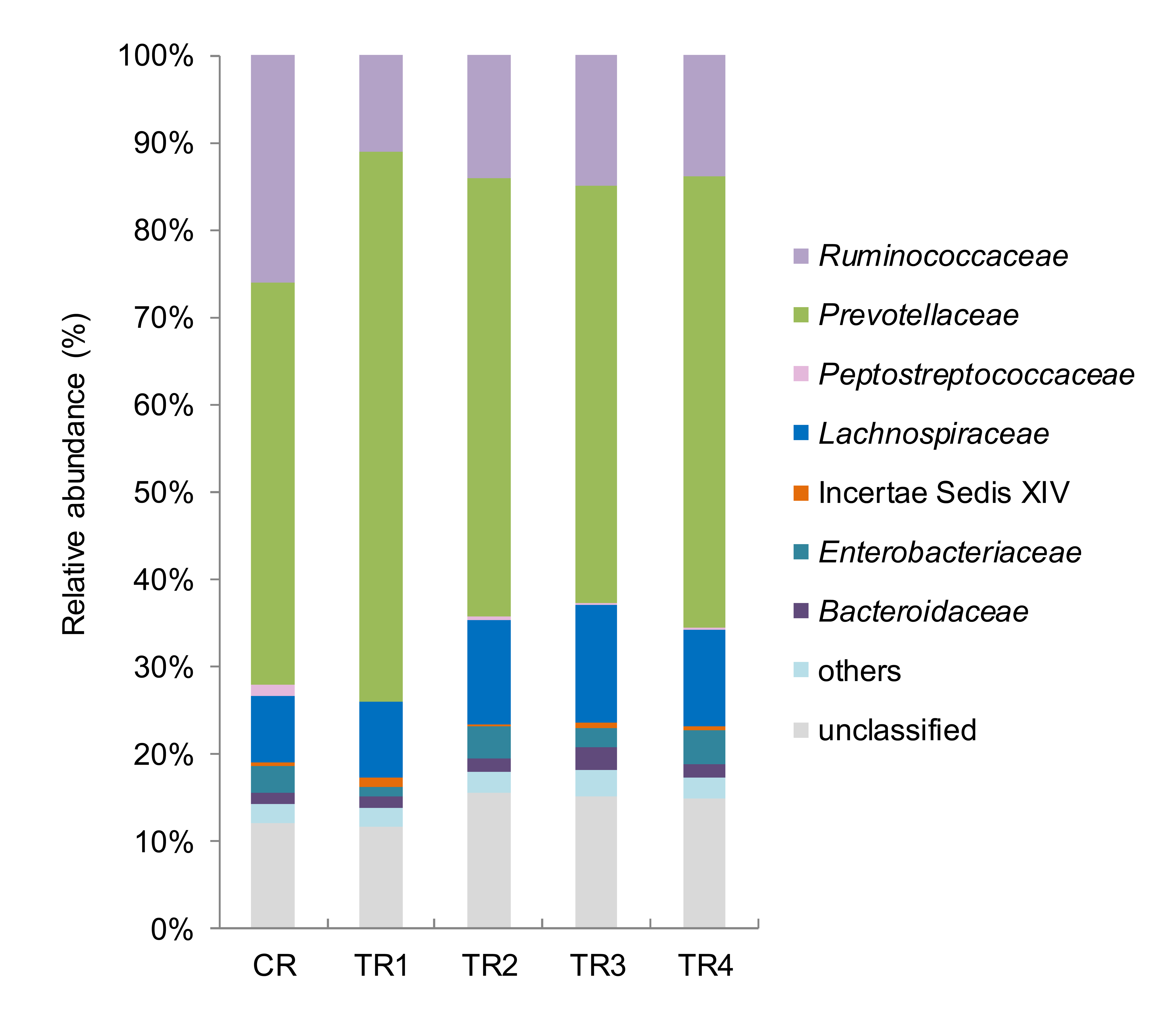

Supplement: Figure S2 — Microbial composition on family level in CR and TR1-4 on day 25 (last day of 3rd stabilization period) measured by 454 pyrosequencing. Values <1% are summarized in the group “others”. (TIF) [file pone.0094123.s002.tif]

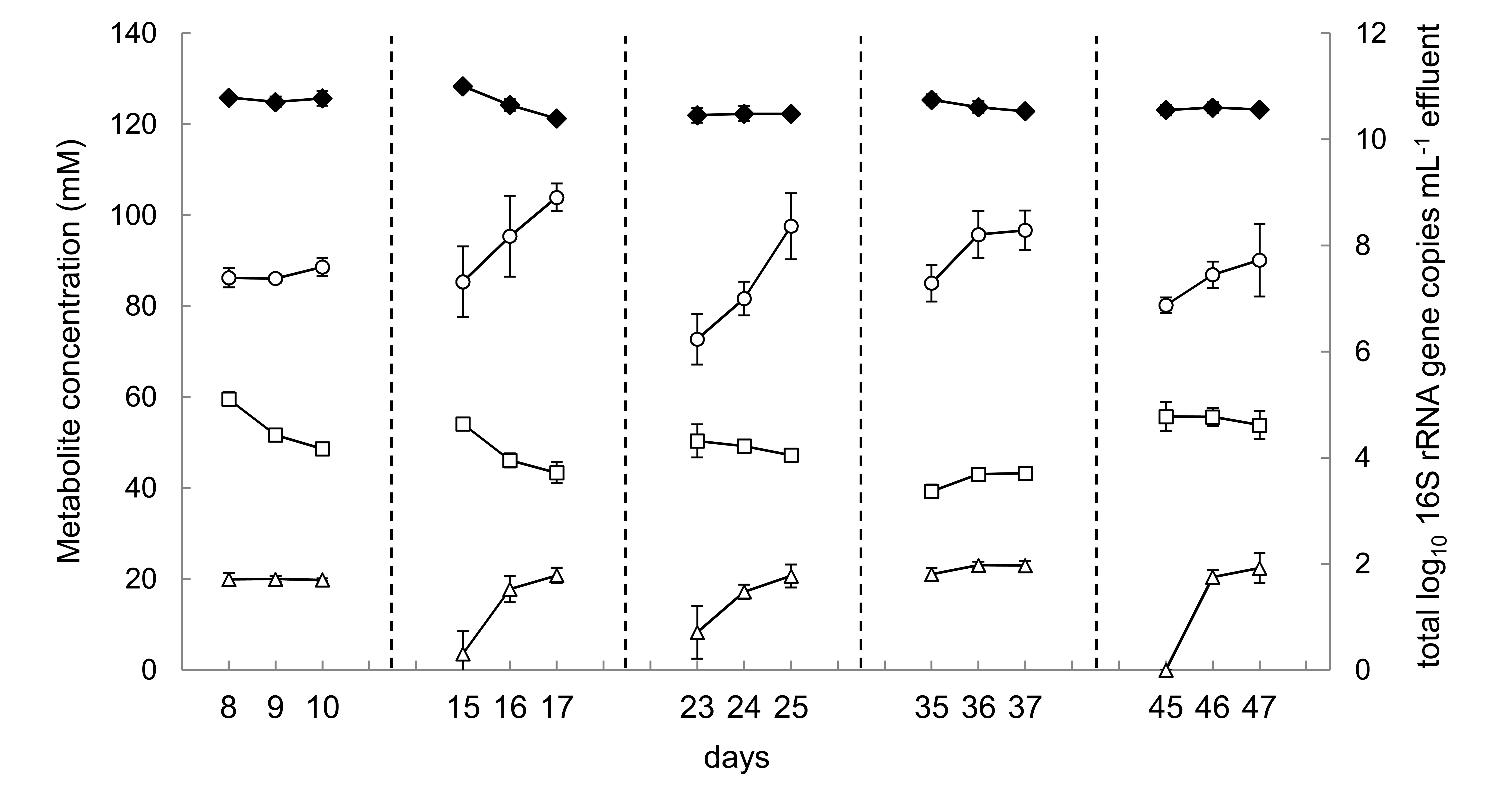

Supplement: Figure S3 — Mean main metabolite concentrations and total 16S rRNA gene copy numbers from TR1-4 during the last three days of each stabilization period. Data are depicted as mean values ± SD from TR1-4. (♦) total 16S rRNA gene copies mL−1 effluent; (○) acetate; (□) propionate; (Δ) butyrate. (TIF) [file pone.0094123.s003.tif]
