# Supplementary material for: In Vitro Continuous Fermentation Model (PolyFermS) of the Swine Proximal Colon for Simultaneous Testing on the Same Gut Microbiota
Source: PLoS One. 2014 Apr 7;9(4):e94123. doi: 10.1371/journal.pone.0094123 (PMC3978012; doi:10.1371/journal.pone.0094123)
Supplement: Table S1 — Composition of the nutritive medium simulating the swine ileal chyme. (PDF) [file pone.0094123.s004.pdf]

Table S1. Composition of the nutritive medium simulating the swine ileal chyme.

Ingredients	g L^{-1}
Carbohydrates	***11.32***
Corn starch	4.32
Pectin (citrus)	2.00
Xylan (beechwood)	2.00
Arabinogalactan (larch wood)	2.00
Guar gum	1.00
N-compounds	***13.00***
Soy Peptone	13.00
Yeast extract	4.50
Mucin (from porcine stomach)	4.00
Salts/Minerals	
L-cysteine HCl monohydrate	0.80
Bile extract porcine	0.40
KH_2PO_4	0.50
$NaHCO_3$	1.50
NaCl	4.50
KCl	4.50
$MgSO_4$ anhy. (120.37 g mol^{-1})	0.64
$CaCl_2 \cdot 2H_2O$ (147.02 g mol^{-1})	0.15
$MnCl_2 \cdot 4H_2O$ (197.91 g mol^{-1})	0.20
Hemin solution (0.05 g mL^{-1})	0.05
Tween 80	1.00
