# Supplementary material for: In Vitro Continuous Fermentation Model (PolyFermS) of the Swine Proximal Colon for Simultaneous Testing on the Same Gut Microbiota
Source: PLoS One. 2014 Apr 7;9(4):e94123. doi: 10.1371/journal.pone.0094123 (PMC3978012; doi:10.1371/journal.pone.0094123)
Supplement: Table S2 — 16S rRNA gene copy numbers (log10 mL−1 of effluent) of specific bacterial groups measured by qPCR in effluent samples from CR and TR1-4. (PDF) [file pone.0094123.s005.pdf]

Table S2. 16S rRNA gene copy numbers (log_{10} mL^{-1} of effluent) of specific bacterial groups measured by qPCR in effluent samples from CR and TR1-4.

		Bifidobacterium **spp.**	***Bacteroides-Prevotella*** **group**	***Enterobacteriaceae***	***Lactobacillus/ Pediococcus/ Leuconostoc*** **spp.**	***Clostridium*** **Cluster IV**
**Stabilization 1**	**CR**	5.4 ± 0.7	10.5 ± 0.1	9.4 ± 0.2	8.4 ± 0.4	9.4 ± 0.3
	**TR1**	5.1 ± 0.2	10.3 ± 0.1	9.5 ± 0.3	8.0 ± 0.2	9.4 ± 0.2
	**TR2**	4.9 ± 0.3	10.4 ± 0.1	9.4 ± 0.3	7.8 ± 0.4	9.5 ± 0.2
	**TR3**	5.6 ± 0.4	10.3 ± 0.2	9.5 ± 0.4	8.1 ± 0.2	9.4 ± 0.3
	**TR4**	4.8 ± 0.4	10.2 ± 0.3	9.5 ± 0.2	8.1 ± 0.3	9.3 ± 0.1
**Stabilization 2**	**CR**	5.9 ± 0.3	10.4 ± 0.1	9.6 ± 0.03	8.0 ± 0.2	10.0 ± 0.1
	**TR1**	6.0 ± 0.7	10.5 ± 0.2	9.8 ± 0.1	7.9 ± 0.5	9.9 ± 0.1
	**TR2**	6.0 ± 0.6	10.5 ± 0.1	9.8 ± 0.04	8.4 ± 0.6	10.0 ± 0.2
	**TR3**	5.8 ± 0.8	10.4 ± 0.1	9.6 ± 0.1	7.6 ± 0.7	9.8 ± 0.1
	**TR4**	6.0 ± 0.6	10.4 ± 0.2	9.8 ± 0.1	7.8 ± 0.6	9.9 ± 0.2
**Stabilization 3**	**CR**	6.1 ± 0.1	10.3 ± 0.1	9.7 ± 0.1	8.3 ± 0.1	10.1 ± 0.1
	**TR1**	5.8 ± 0.4	10.5 ± 0.1	9.5 ± 0.04	8.7 ± 0.3	9.9 ± 0.3
	**TR2**	6.0 ± 0.4	10.5 ± 0.2	9.9 ± 0.1	8.8 ± 0.5	9.9 ± 0.2
	**TR3**	5.8 ± 0.2	10.4 ± 0.1	9.7 ± 0.1	8.3 ± 0.6	9.8 ± 0.2
	**TR4**	5.9 ± 0.3	10.3 ± 0.1	9.8 ± 0.1	8.7 ± 0.6	9.8 ± 0.1
**Stabilization 4**	**CR**	6.6 ± 0.1	9.9 ± 0.1	9.9 ± 0.03	7.7 ± 0.3	10.0 ± 0.1
	**TR1**	7.3 ± 0.1	10.0 ± 0.2	9.8 ± 0.1	8.4 ± 0.2	10.1 ± 0.1
	**TR2**	6.4 ± 0.1	9.9 ± 0.1	9.9 ± 0.3	7.8 ± 0.1	10.1 ± 0.1
	**TR3**	6.5 ± 0.04	10.0 ± 0.1	10.1 ± 0.2	8.2 ± 0.3	10.2 ± 0.2
	**TR4**	6.3 ± 0.1	10.0 ± 0.1	9.9 ± 0.2	8.0 ± 0.4	10.2 ± 0.1
**Stabilization 5**	**CR**	6.7 ± 0.2	10.0 ± 0.2	9.9 ± 0.02	8.4 ± 0.2	9.8 ± 0.3
	**TR1**	7.7 ± 0.6	10.1 ± 0.1	9.8 ± 0.1	8.7 ± 0.4	9.6 ± 0.1
	**TR2**	6.3 ± 0.7	10.0 ± 0.1	9.9 ± 0.2	8.6 ± 0.5	9.7 ± 0.3
	**TR3**	6.5 ± 0.6	10.0 ± 0.1	9.9 ± 0.1	8.8 ± 0.5	9.7 ± 0.2
	**TR4**	6.6 ± 0.6	10.1 ± 0.1	10.0 ± 0.1	8.7 ± 0.5	9.9 ± 0.3

Data are log_{10} transformed and mean values for the last three days of each stabilization period. No significant differences were observed for all time points tested using the Mann-Whitney U test ($P<0.05$)
